# Gaidai-Xing reliability method validation for 10-MW floating wind turbines

**DOI:** 10.1038/s41598-023-33699-7

**Published:** 2023-05-29

**Authors:** Oleg Gaidai, Yihan Xing, Jingxiang Xu, Rajiv Balakrishna

**Affiliations:** 1 Shanghai Ocean University, Shanghai, China; 2 Department of Mechanical and Structural Engineering and Materials Science, University of Stavanger, Stavanger, Norway

**Keywords:** Energy science and technology, Engineering, Mathematics and computing

## Abstract

In contrast to well-known bivariate statistical approach, which is known to properly forecast extreme response levels for two-dimensional systems, the research validates innovative structural reliability method, which is particularly appropriate for multi-dimensional structural responses. The disadvantage of dealing with large system dimensionality and cross-correlation across multiple dimensions is not a benefit of traditional dependability approaches that deal with time series. Since offshore constructions are built to handle extremely high wind and wave loads, understanding these severe stresses is essential, e.g. wind turbines should be built and operated with the least amount of inconvenience. In the first scenario, the blade root flapwise bending moment is examined, whereas in the second, the tower bottom fore-aft bending moment is examined. The FAST simulation program was utilized to generate the empirical bending moments for this investigation with the load instances activated at under-rated, rated, and above-rated speeds. The novel reliability approach, in contrast to conventional reliability methods, does not call for the study of a multi-dimensional reliability function in the case of numerical simulation. As demonstrated in this work, it is now possible to assess multi-degree-of-freedom nonlinear system failure probability, in the case when only limited system measurements are available.

## Introduction

Developing more efficient wind turbines is a driving force, enabling engineers to achieve net-zero emissions target 2050^1^. According to International Electrotechnical Commission (IEC) standards, wind turbines must be designed to operate in the highly stochastic wind and wave environments for at least 20 years^2^. Since both larger and more wind turbines are constructed, especially offshore, it has become extensional to minimise construction, maintenance, and operational costs. Turbines and their components are vulnerable to various cyclic loads such as axial and transverse loading, twisting moments and torque. Furthermore, the loads acting on the wind turbines are also influenced by the wind's stochastic behaviours in speed, direction, shear, and vorticity, making fatigue damge analysis imperative for wind turbines design and operation^3–6^. Any failure in the turbine system can result in unnecessary downtime, which can be extremely expensive^7–9^. Despite this, engineers thought extensive modeling wasn't essential in the 1970s, leading to the building of wind turbines with enormous safety margins. This, however, changed as larger wind turbines continued to emerge since it became more expensive to maintain comparable safety margins. Additionally, incorrect design load estimation resulted in unneeded failures. These prompted an overhaul of the industry, and by the 1990s, control algorithms, turbulence models, dynamic structural models, and aerodynamic models had all been combined to provide a more accurate forecast approach^10–14^.

In^15^ authors conducted uncertainty analysis while continuing to fine-tune parametric models and probabilistic approaches. While attempting to obtain a more precise estimation at this time, in^16–18^ authors attempted to compile and simplify the different techniques mentioned above. Many studies have recently focused on more precise estimation of the wind turbine’s damage^19–26^. There is a practical engineering need to apply robust statistical techniques that tackle limited data sets, and estimate reasonable and accurate structural damage. There is also a need to investigate the statistical accuracy of these techniques in more detail, and develop new improved methods suitable for limited non-stationary data sets^27–33^. If available system response time series are relatively short, the advocated Monte Carlo based approach becomes especially attractive.

### System description

**Figure 1:**
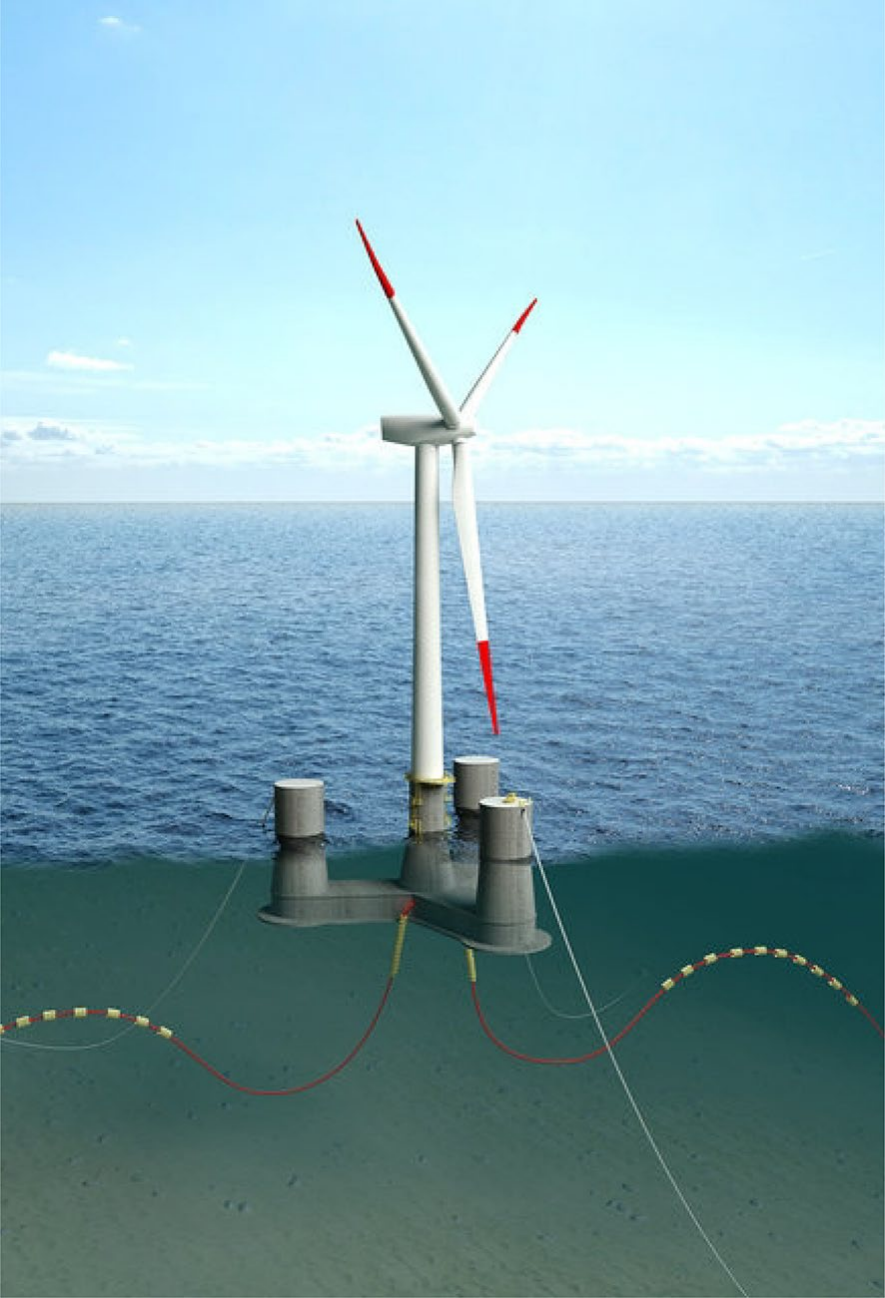
The 10-MW OO-Star floating wind turbine^37^.

A 10-MW FWT system^34–37^ is used in this work, which is illustrated in Fig. 1. The FWT system will be expounded in two parts in the following sections. Firstly, the reference wind turbine will be described, then the properties of the semi-submersible floater and the mooring system will be introduced.

### DTU 10-MW reference wind turbine

In this study, a 10-MW reference wind turbine (RWT) built from the NREL 5-MW RWT is employed. The wind turbine is a conventional three-bladed, clockwise rotation-upwind turbine that is outfitted with a variable speed and collective pitch control system. It was developed in accordance with the International Electrotechnical Commission (IEC) Class 1A wind regime. Numerous academic papers have successfully constructed and researched the DTU 10-MW RWT numerical model^38–43^. The summary of the DTU 10-MW RWT is shown in Table 1.

**Table 1:** DTU 10-MW reference wind turbine key parameters^37^.

Parameter	Value
Rating	10-MW
Type	Upwind/3 blades
Control	Variable speed, collective pitch
Drivetrain	Medium-speed, multiple stage gearbox
Cut-in, rated and cut-out wind speed (m/s)	4, 11.4, 25
Minimum and maximum rotor speed (rpm)	6.0, 9.6
Maximum generator speed (rpm)	480
Rotor diameter (m)	178.3
Hub height (m)	119.0
Rotor mass (kg)	227962
Nacelle mass (kg)	446036
Tower mass (kg)	^1.257^10^6^

### OO-Star semi-submersible wind floater and mooring system

**Figure 2:**
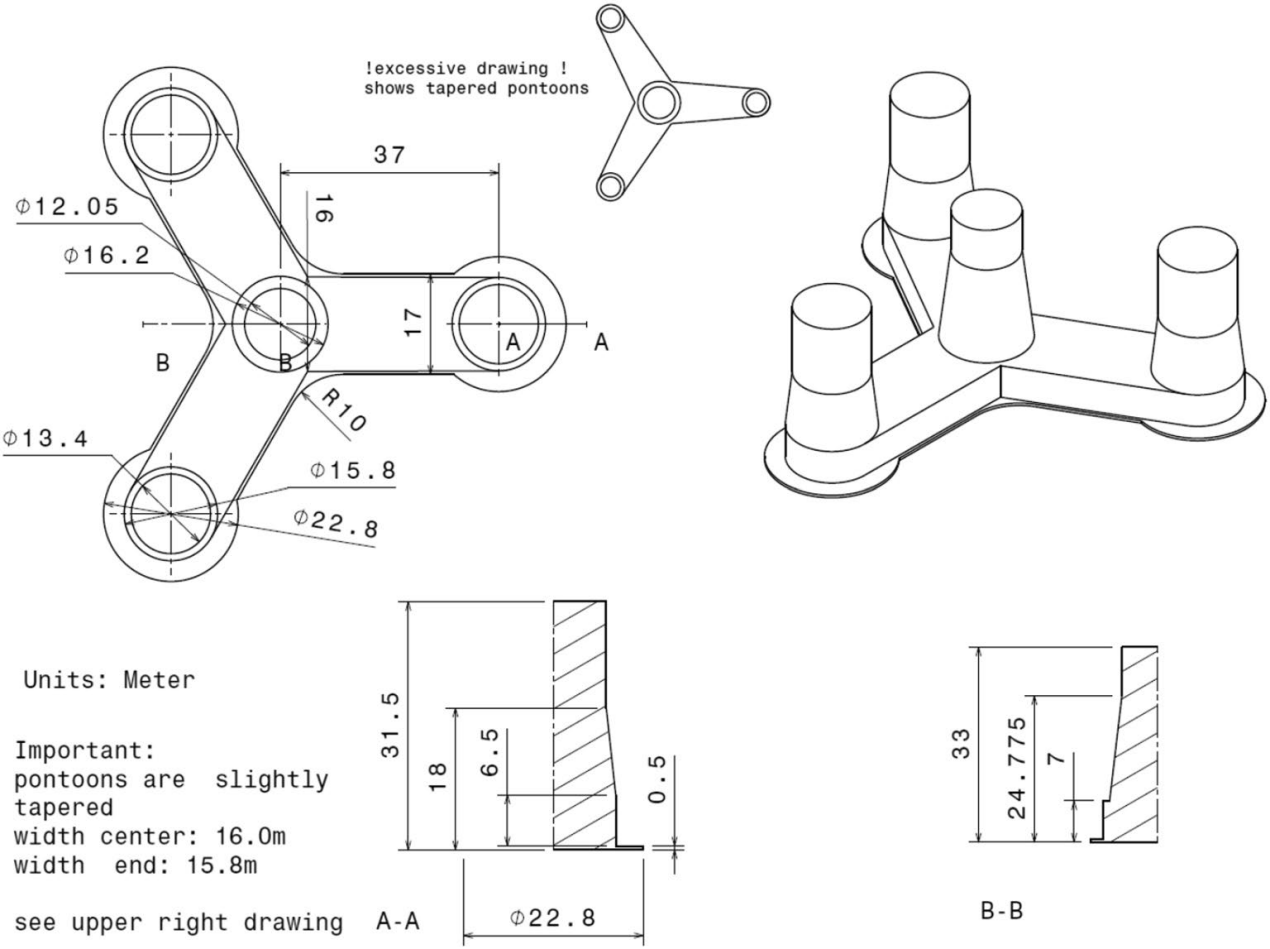
Main dimensions of the OO-Star floater of the 10-MW wind turbine.

This work uses a semi-submersible floating structure to support the 10-MW RWT. It was introduced in^37^ in the LIFES 50 + project^37^. The floater comprises post-tensioned concrete, hosting a central column with three outer columns. The four columns are mounted on a star-shaped pontoon, where a slab is attached at the bottom. Three catenary mooring lines are used to maintain the floater in position, and in each line, a clumped mass is attached, separating the line into two segments. Greater details of the OO-Star Wind Floater and the mooring system are shown in Fig. 2, Tables 2 and 3, respectively.

**Table 2:** The main properties for the 10-MW OO-Star wind floater.

Parameter	Value
Water depth (m)	130
Draft (m)	22
Tower-base interface above mean sea level (m)	11
Displacement (kg)	24,158
Overall gravity, including ballast (kg)	21,709
Roll and pitch inertia about center of gravity (kg∙m^2^)	1.4462 × 10^10^
Yaw inertia about center of gravity (kg∙m^2^)	1.63 × 10^10^
Center of gravity height below mean sea level (m)	15.23
Center of buoyancy height below mean sea level (m)	14.236

**Table 3:** The main properties for the mooring system of the 10-MW FWT.

Parameter	Value
Radius to anchors from platform centerline (m)	691
Anchor position below MSL (m)	130
Initial vertical position of clump mass below MSL (m)	90.45
Initial radius to clump mass from centerline (m)	148.6
Equivalent weight per length in water (N/m)	3200.6
Extensional stiffness (N/m)	1.506 × 10^9^

**Figure 3:**
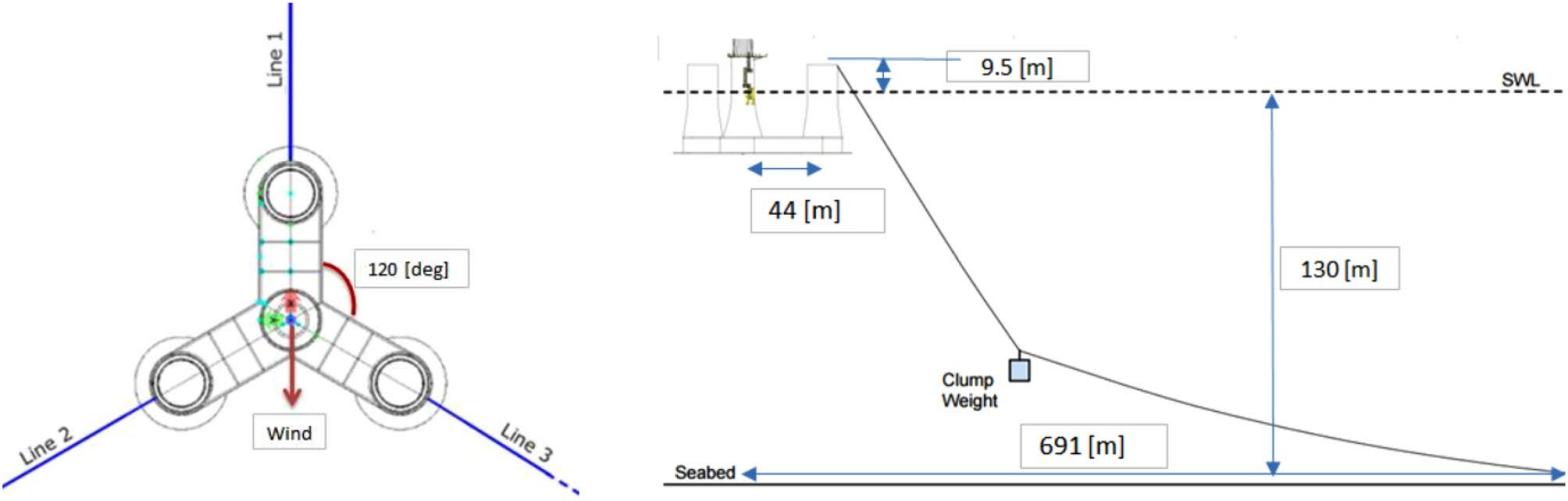
Sketch of 10-MW FWT mooring system (left: top view; right: side view).

Fatigue, Aerodynamics, Structures and Turbulence (FAST) (version8, v8.16.00a-bjj), an open-source WT simulation tool developed by the National Renewable Energy Laboratory (NREL), is utilized in this work for the fully coupled aero-hydro-elastic-servo dynamic analysis for the 10-MW FWT, Fig. 3. The FAST code couples together five computer codes: AeroDyn^44^, HydroDyn^45^, ServoDyn, and MoorDyn^46^, must take into consideration the dynamics of the mooring system, control dynamics, structural dynamics, hydrodynamic loads on floaters, and aerodynamic stresses on rotor blades. In addition, FAST offers the interface for reading the time-varying stochastic wind for time-domain simulations. Other well-known projects like have utilized the FAST simulation tool with success, OC3: Offshore Code Comparison Collaboration^47^ and OC4: IEA Task Wind 30^48^, and its modelling capability has been authenticated, using multiple floating structures in the Netherlands^49^.

#### Aerodynamics

The Blade Element Momentum (BEM) hypothesis was used to determine the aerodynamic loads on the blades. Blade element theory and momentum theory were combined in BEM theory. The BEM approach incorporates a number of sophisticated adjustments, such as tip loss, hub loss, skewed inflow, and dynamic stall corrections. In order to account for the hub and blade tip losses brought on by a limited number of blades, Prandtl adjustments were used. The induction factors are taken into account using the Glauert correction, while the skewed inflow correction is taken into account using the Pitt and Peters' model. The Beddoes-Leishman model made use of the dynamic stall correction. The AeroDyn theory documentation has more information on the FAST code's aerodynamic load computation^44^.

#### Hydrodynamics

Based on potential flow theory and taking into account Morison's drag term, the hydrodynamic loads acting on the semi-submersible floater are estimated. It takes into consideration, respectively, the viscous loads and wave pressures. According to the potential flow theory, a panel code called WAMIT estimates hydrodynamic coefficients like additional mass and potential damping coefficients and the first-order wave excitation load transfer function in the frequency domain first. The convolution method is then used to translate these hydrodynamic coefficients into the time domain.

#### Structural dynamics

The FAST code takes into consideration the structural dynamics of the FWT’s structural dynamics of the FWT’s structural dynamics of the FWT’s structural dynamics of the FWT’s structural dynamics of the FWT’s structure The nacelle, hub, and floater are hard bodies, but the blades, tower, and driveshaft are regarded as flexible bodies. The Rayleigh damping model is used to depict the inbuilt structural damping in the blades and tower. When using Kane's method to derive the rigid-flexible coupled system, the equations of motion are solved to determine the structural dynamic responses in the time domain^50^.

#### Control system dynamics

The below-rated and full-rated regions are the two operational modes of the control system employed in the 10-MW FWT. In the below-rated zone, the generator torque-speed curve controls the rotor rotational speed with the best tip speed ratio in order to generate the most power. The blade pitch angle is controlled by a proportional-integral (PI) algorithm to lessen structural loads while maintaining rated power output in the complete rated zone. To prevent the detrimental damping effects, which are crucial in altering the platform movements for FWTs, the PI parameters from the land-based RWT are changed.

**Figure 4:**
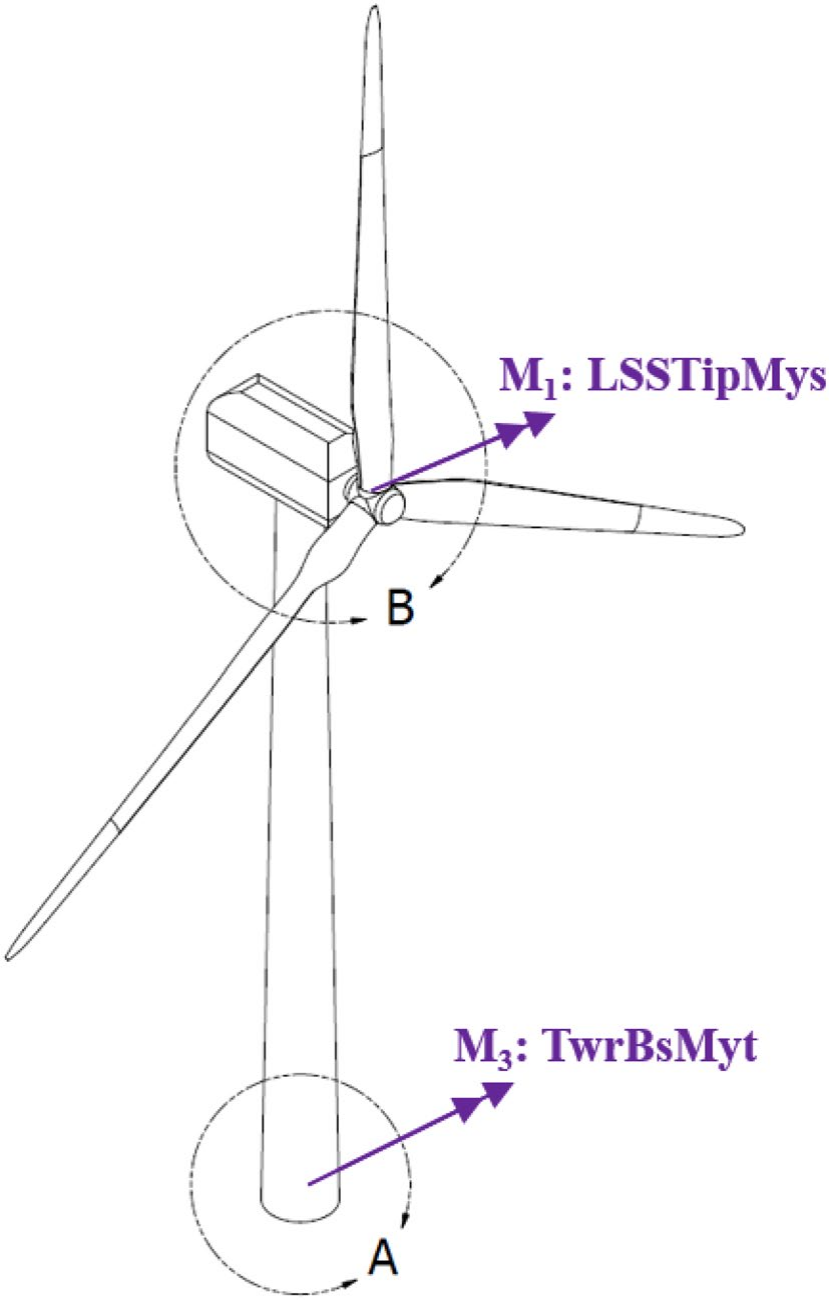
Location of points where bending moments and stresses are measured.

The loads at the two places of measurement shown in Fig. 4 are considered. These are the blade 1 root flapwise bending moment (RootMyb1) along with tower bottom fore-aft bending moment (TwrBsMyt).

Figure 4 presents Location of points where FWT bending moments and stresses are measured. These are the blade 1 root flapwise bending moment (RootMyb1) and tower bottom fore-aft bending moment (TwrBsMyt).

### Load cases and environmental conditions

The environmental data (wind and wave data) used in this paper are established based on hindcast data from an offshore site in the Northern North Sea from 2001 to 2010. The long-term joint wind and wave distribution were developed in^51^, which considers a one-hour mean wind speed at the position that is 10 m above the sea level (*U*_*10*_), wave spectral peak period (*T*_*p*_) and the significant wave height (*H*_*s*_). The joint distribution of *U*_*10*,_ *H*_*s*_ and *T*_*p*_ is expressed as below:

1 $${f}_{{U}_{10,}{H}_{s,}{T}_{p}}\left(u,h,t\right)={f}_{{U}_{10}}\left(u\right)\cdot {f}_{{H}_{s}{|U}_{10}}\left(h|u\right)\cdot {f}_{{T}_{p}{|U}_{10, }{H}_{s}}\left(t|u,h\right),$$

where ${f}_{{U}_{10}}(u)$ *, *${f}_{{H}_{s}{|U}_{10}}(h|u)$* and* ${f}_{{T}_{p}{|U}_{10, }{H}_{s}}\left(t|u,h\right)$ represents the marginal distribution of *U*_*10*_, the conditional distribution of *H*_*s*_ for given *U*_*10*_ and the conditional distribution of *T*_*p*_ for given *U*_*10*_ and *H*_*s*_.

Three representative load cases with a high probability of occurrence in the normal operating conditions are used in the present work and listed in Table 4. The mean wind speed selected to be used in this paper is based on the turbines operating ranges (wind speeds ranging within the cut-in, rated and cut-out zones) with an increment size of 4 m/s. The most probable wave height and spectra peak period in each wind speed condition is selected based on the joint distribution expressed in Eq. (1).

**Table 4:** Load cases for numerical simulations.

Load cases	${U}_{w}$ (m/s)	${T}_{I}$	${H}_{s}$(m)	${T}_{p}$(s)	Samples	Simulation length (s)
LC1	8	0.1740	1.9	9.7	20	4000
LC2	12	0.1460	2.5	10.1	20	4000
LC3	16	0.1320	3.2	10.7	20	4000

Modelled turbulent wind and irregular waves are taken into account in all load scenarios to be directionally aligned. The wind turbine Class C is used, and the normal turbulence and normal wind profile models are used. The wind speed profile is modelled using the wind power-law formulation

2 $${U}_{w}\left(z\right)= {{U}_{hub} (\frac{Z}{{Z}_{hub}})}^{\alpha },$$

where *U*_*w*_*(z)* is the mean wind speed at the height $z$ above the still water level, *u*_*hub*_ represents the mean wind speed at the hub height, *z*_*hub*_ denotes the hub height above the still water level and is 119 m for the 10-MW FWT. *α* is the power-law exponent, and it is taken as 0.14 for offshore locations based on the recommendation in IEC 61400-3-2^52^.

The Kaimal turbulence model is used to generate the three-dimensional turbulent wind fields, simulated using a stochastic turbulent-wind simulator, Turbsim^53^. Time-varying irregular waves are generated using the Joint North Sea Wave Project (JONSWAP) spectrum according to the specified *H*_*s*_ and *T*_*p*_. Detailed descriptions for the models of turbulent wind and irregular waves can be found in IEC 61400-3-2^52^.

Twenty separate random samples of wind and wave are applied for each sea state for each of the three environmental variables. Each simulation lasts for 4000 s, with the first 400 s being omitted to lessen the transitory effect brought on by the start-up of the wind turbine. Therefore, 1-h data in each simulation is created and is used for extreme value analysis in this study. To limit the stochastic unpredictability, the results in this work are based on an average of 20 1-h simulations.

## Gaidai-Xing method

Using traditional engineering reliability methods to estimate structural system reliability is difficult^50,54–61^. The latter is frequently brought on by a great deal of system freedom and random factors that regulate dynamic systems. A complicated structural system’s reliability can be directly determined by doing direct numerical Monte Carlo simulations or by having adequate observations. However, for many complicated engineering dynamic systems, computational and experimental methods are frequently out of reach. The authors' unique dependability technique for structural systems aims to lower the expenses associated with measurement.

**Figure 5:**
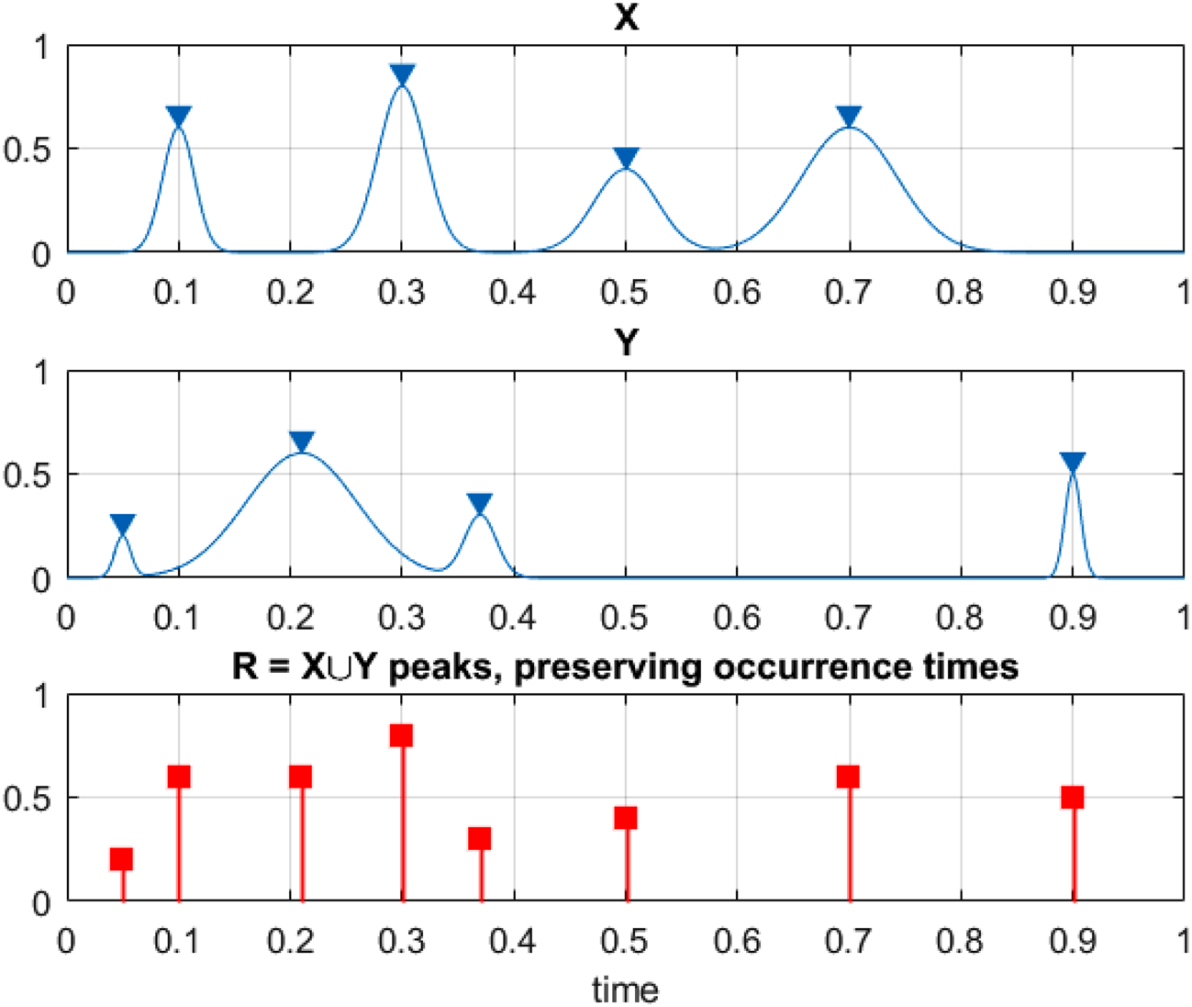
Example of how 2 components, X and Y, being merged to create 1 new synthetic vector $\overrightarrow{R}$.

Typically, it is considered that ocean waves follow an ergodic random process (stationary and homogenous). Consider a structure with several degrees of freedom that is exposed to random ergodic environmental loadings that are stationary in time, such as wind and waves from the environment. Let one consider multi degree of freedom (MDOF) structural dynamic either response or load, or combined system components vector $\left(X\left(t\right), Y\left(t\right), Z\left(t\right), \dots \right)$, that has been either measured or simulated over a sufficiently long time period $(0,T)$. Unidimensional system component vector global maxima being denoted as ${X}_{T}^{\mathrm{max}}=\underset{0\le t\le T}{\mathrm{max}}X\left(t\right)$, ${Y}_{T}^{\mathrm{max}}=\underset{0\le t\le T}{\mathrm{max}}Y\left(t\right)$, ${Z}_{T}^{\mathrm{max}}=\underset{0\le t\le T}{\mathrm{max}}Z\left(t\right), \dots$. By sufficiently long time period $T$ authors mean large enough value of $T$ with respect to the dynamic system auto-correlation and relaxation times. Let ${X}_{1},\dots ,{X}_{{N}_{X}}$ be temporally consequent local maxima of the component process $X=X(t)$ at discrete temporally increasing times ${t}_{1}^{X}<\dots <{t}_{{N}_{X}}^{X}$ within $(0,T)$. Identical definitions follow for other MDOF components $Y\left(t\right), Z\left(t\right), \dots$ namely ${Y}_{1},\dots ,{Y}_{{N}_{Y}};$ ${Z}_{1},\dots ,{Z}_{{N}_{Z}}$ and so on. For simplicity, all system components, and hence their maxima have been assumed to be non-negative^59,62–70^. Then

3 $$P=\underset{\left(0, 0, 0, , \dots \right)}{\overset{\left({\eta }_{X}, {\eta }_{Y}, {\eta }_{Z }, \dots \right)}{\iiint }}{p}_{{X}_{T}^{\mathrm{max}}, { Y}_{T}^{\mathrm{max}}, { Z}_{T}^{\mathrm{max}} , \dots }\left({x}_{T}^{\mathrm{max}}, {y}_{T}^{\mathrm{max}},{ z}_{T}^{\mathrm{max}}, \dots \right)d{x}_{T}^{\mathrm{max}}d{y}_{T}^{\mathrm{max}}d{z}_{T}^{\mathrm{max}},\dots$$

being the probability of dynamic system survival with critical values of system components being denoted as ${\eta }_{X}$, ${\eta }_{Y}$, ${\eta }_{Z}$,…; $\cup$ being logical unity operator «or»; ${p}_{{X}_{T}^{\mathrm{max}}, { Y}_{T}^{\mathrm{max}}, { Z}_{T}^{\mathrm{max}} , \dots }$ being joint probability density function (PDF) of the individual component maxima. Is system number of degrees of freedom (NDOF) is large, it is not practically feasible to estimate directly the joint PDF ${p}_{{X}_{T}^{\mathrm{max}}, { Y}_{T}^{\mathrm{max}}, { Z}_{T}^{\mathrm{max}} , \dots }$ and therefore survival probability $P$. The latter probability $P$ however, needs to be estimated, as system expected lifetime, according to Eq. (1). Bio-system unidimensional components $X, Y, Z, \dots$ being now re-scaled and non-dimensionalized as follows

4 $$X\to \frac{X}{{\eta }_{X}}, Y\to \frac{Y}{{\eta }_{Y}}, Z\to \frac{X}{{\eta }_{X}}, \dots$$

making all two responses non-dimensional and having the same failure limit equal to 1. Next, unidimensional system components local maxima being merged into one temporally non-decreasing synthetic vector $\overrightarrow{R}=\left({R}_{1}, {R}_{2}, \dots ,{R}_{N}\right)$ in accordance with corresponding merged time vector ${t}_{1}\le \dots \le {t}_{N}$, $N\le {N}_{X}+{N}_{Y}+{ N}_{Z}+\dots$. Each local maxima ${R}_{j}$ being actual encountered dynamic system component local maxima, corresponding to either $X\left(t\right)$ or $Y\left(t\right)$, or $Z\left(t\right)$ or other system components^71–73^. Constructed synthetic $\overrightarrow{R}$-vector has no data loss, see Fig. 5.

Now the non-decreasing synthetic vector $\overrightarrow{R}$, and it’s corresponding temporally non-decreasing occurrence times ${t}_{1}\le \dots \le {t}_{N}$, have been fully introduced.

Distinctive feature of Gaidai-Xing method is that it is uning deconvolution method to perform numerically accurate and stable extrapolation^30,31^.

An obvious limitation of the suggested method lies within underlying system stationarity assumption. Suggested methodology can tackle non-stationary systems (for example systems with degradation) as well, provided representative system observation sample is present and the underlying trend is known.

## Results

This paper presents the methodology for estimating the 10 MW DTU WT-OO-Star's extreme loads during operating conditions. The empirical data is based on accurate numerical simulations using a FAST model as presented in Sect. “Introduction”. The Gaidai-Xing method is presented in Sect. “Gaidai-Xing method”. The proposed methodology provides proper bivariate extreme value prediction, utilizing all available data efficiently. Based on the overall performance of the proposed method, it was concluded that the bivariate modified Weibull method could incorporate environmental input and provide a more robust bivariate prediction based on proper numerical simulations. The described approach may be used at the design stage of a large FWT to provide the opportunity of defining FWT parameters that would minimize extreme loads and potential damages.

This section presents statistical analysis results for M_1_ and M_3_ bending moments. The focus is on accurate predicting extreme response, which is vital for safety and reliability at the design stage. The conditioning level *k* is set to be 10, as it was observed that ACER functions have converged at that level in the distribution tail.

**Figure 6:**
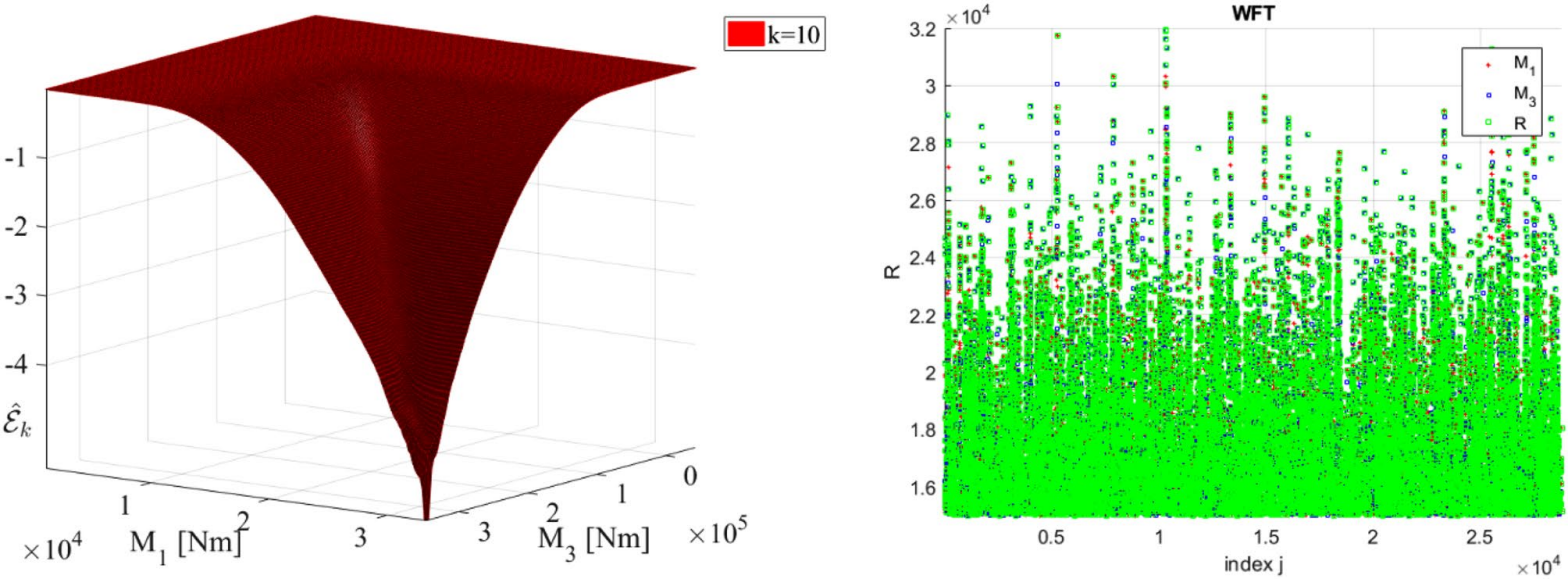
Left: bivariate empirical bivariate modified Weibull function ${\widehat{\mathcal{E}}}_{k}$, decimal log scale. Right: non-dimensional $R\left(t\right)$, presented as time series.

Figure 6 left presents the phase space for responses M_1_ vs M_3_, along with the bivariate empirical bivariate modified Weibull function ${\widehat{\mathcal{E}}}_{k}$^27,62,74–76^. Figure 6 right presents non-dimensional ${\varvec{R}}\left(t\right)$ from Sect. “Gaidai-Xing method”, presented as time series.

This section illustrates efficiency of Gaidai-Xing method, by means of application to WFT bending moments data set. Two different WFT bending moments M_1_ and M_3_ were chosen as components $X, Y$ thus constituting an example of two dimensional (2D) dynamic system. In order to unify both measured time series $X, Y$ the following scaling was performed according Eq. (4) making both two responses non-dimensional and having the same failure limit equal to 1. Next, all local maxima from both measured time series were merged into one single time series by keeping them in time non-decreasing order: $\overrightarrow{R}=\left(\mathrm{max}\left\{{X}_{1},{Y}_{1}\right\},\dots ,\mathrm{max}\left\{{X}_{N},{Y}_{N}\right\}\right)$. In order to unify both measured time series $X, Y$ the following scaling was performed according Eq. (2).

**Figure 7:**
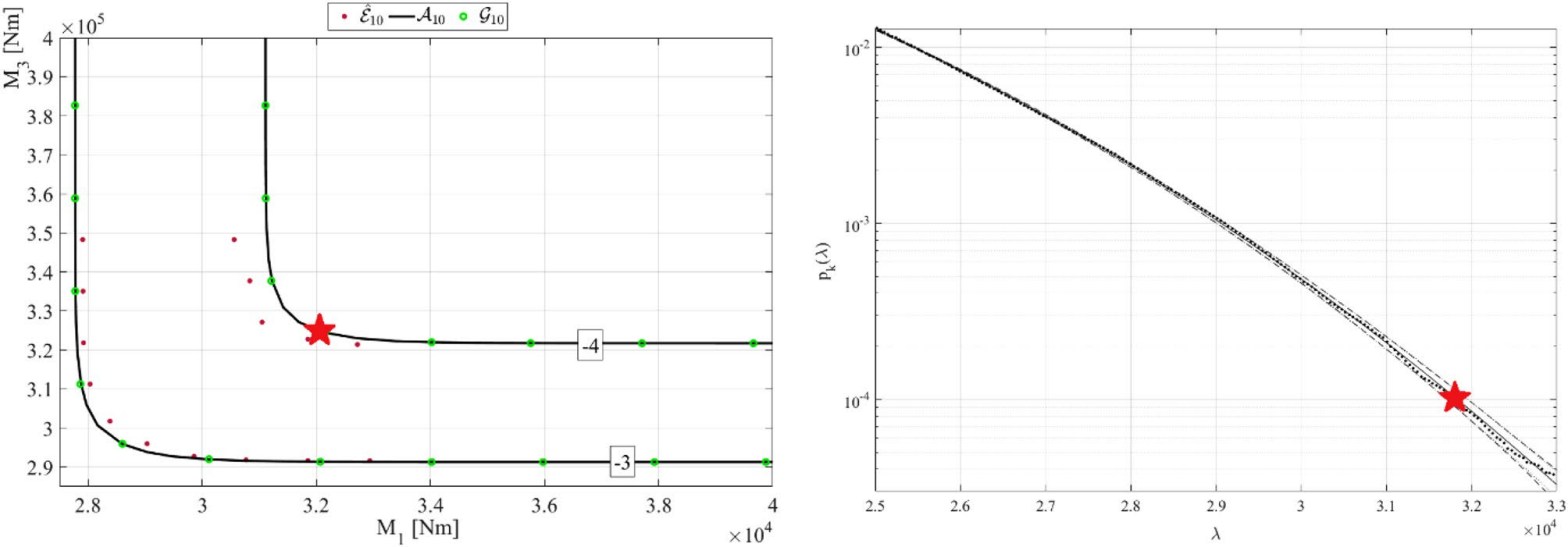
Left: bivariate modified Weibull bivariate contours for WFT non-dimensional bending moments, right: Gaidai-Xing prediction. Star indicates the same bivariate failure level of interest.

Figure 7 presents bivariate modified Weibull bivariate contours for WFT bending moments. It is seen from Fig. 7 left that bivariate modified Weibull fits different Gumbel copula to the measured data, and there is an inherent error due to particular copula choice. For more details on bivariate modified Weibull method^53,59,62–70^. Bivariate non-dimensional failure point indicated by star in Fig. 7 was chosen. The probability level $p={10}^{-4}$ corresponding to this contour line was then compared with Gaidai-Xing method estimate. It was found that bivariate modified Weibull probability level estimate lied well within 95% CI (Confidence Interval), predicted by Gaidai-Xing method.

## Conclusions

Traditional time series-based dependability techniques lack the ability of effectively dealing with highly dimensional systems and cross-correlation between various system responses. The principal benefit of the methodology is its ability to analyze the dependability of high-dimensional dynamic systems.

In this study, both the dynamic response of the simulated WFT and a synthetic wind speed data set were evaluated. The theoretical justification for the proposed technique is explained in depth. Notably, although using direct measurement or Monte Carlo simulation to analyze the reliability of dynamic systems is appealing, the complexity and high dimensionality of dynamic systems necessitate the development of novel and robust techniques that can handle the available data and utilize it as efficiently as possible.

The methodology outlined in this study has already been demonstrated to be effective when applied to a variety of simulation models, but only for one-dimensional system responses. Typically, extremely precise predictions were made. The objective of this study was to develop a general-purpose, dependable, and user-friendly multidimensional dependability approach. To sum up, the recommended technique can be applied to a diversity of engineering disciplines. In no way does the provided example of naval architecture limit the potential applications of a novel method.

## Data Availability

Data available on request from the corresponding author.

## Abbreviations

FAST: Simulation tool, developed by the national renewable energy laboratory
FWT: Floating wind turbine
DTU: Danish technical university
RWT: DTU 10-MW reference wind turbine
Bivariate modified Weibull: Bivariate averaged conditional exceedance rate
